# Examining drivers of the emissions embodied in trade

**DOI:** 10.1371/journal.pone.0176159

**Published:** 2017-04-20

**Authors:** Leying Wu, Zheng Wang

**Affiliations:** 1 Key Laboratory of Geographic Information Science, East China Normal University, Ministry of Education, Shanghai, China; 2 Institute of Policy and Management, Chinese Academy of Sciences, Beijing, China; Beijing Normal University, CHINA

## Abstract

Emissions embodied in provincial trade (EEPT) have important effects on provinces’ responsibilities for carbon emission reductions. Based on a multi-regional input-output model, we calculated EEPT for China’s 30 provinces in 2002, 2007 and 2010, and we attempted to determine the drivers of EEPT. The results showed that, during this period, the ratio of EEPT to production-based emissions increased over time, reaching 40.24% in 2010. In consideration of its important role in carbon emissions, we analyzed the factors attributable to EEPT through structure decomposition analysis. The decomposition results showed that final demand and carbon emission intensity were two major factors in EEPT, while the final demand in other provinces and the carbon emission intensity in the local province were major factors for Emissions embodied in provincial exports and the final demand in the local province and the carbon emission intensity in other provinces were major factors for Emissions embodied in provincial imports. Regarding the differences among the EEPT of different provinces, changes in the structure of trade were the primary reason.

## Introduction

China has become the largest emissions emitter in the world in 2006 [1]. The Chinese government has put forward itsown reduction goal, which is to reduce the carbon intensity of GDP by 40–45 percent compared with that in 2005. During the China-US joint statement on climate change, the Chinese government claimed that the carbon emission would peak at 2030 or earlier. Therefore, the reductions of carbon emissions have been an urgent priority for the Chinese government. To realize this reduction, clarifying the differentiated commitments of various provinces has been particularly critical [2, 3]. However, with the rapid development of economic globalization and the geographic separation of consumers, the pollution emitted in the production of consumable items has been caused by regional trade, indicating that there would be significant differences between the carbon emissions occurring from the region’s economic production and the carbon emissions stemming from economic consumption (direct and indirect) within the region [4]. These differences make the emissions embodied in regional trade (EEPT) essential to regional reduction responsibilities.

As the world’s factory and the largest exporter in the world, China has emissions driven in large part by other countries. Peters and Hertwich[5] found that China’s carbon emissions based on production (3289.2 Mt) were almost 600 Mt larger than that based on consumption, and more seriously, the percentage of emissions embodied in exports compared with total production-based emissions was 24.4% in 2001. Obviously, since China has partly accepted responsibility for reductions for those importing countries when production-based emissions are used, international trade has a substantial impact on China’s reduction responsibility [6, 7]. Therefore, earlier researchers primarily focused on the effects of emissions embodied in exports on China’s reduction policy[8], such as in trade between China and the US[9, 10], China and Japan[11–13], China and the UK[14], and China and the Asian economies[15].

By analyzing EET at the national scale, these previous studies concerning the emissions embodied in international trade provided solid and effective policy recommendations for China to formulate emissions reductions for climate policy. Unfortunately, these analyses paid little attention to discussing the impact of trade on emissions from a regional perspective. In fact, due to provincial diversities in natural resources and industrial infrastructure, reduction targets at the national scale should be achieved through implementations on the provincial scale. Additionally, due to some over-valuation of low carbon intensity regions and undervaluation of high carbon intensity regions, the EET considering China as a whole is larger than that based on the provincial scale [16]. Therefore, the provincial IO model considering interprovincial trade is more suitable for EET studies in China [17, 18]. Actually, provincial emissions are significantly different in China[19], and production has been transferred from less developed regions to developed regions, leading emissions transitions from the base of the energy and heavy chemical industries to developing regions and undeveloped regions with incomplete industrial infrastructures[20–22], and as a result, the emissions embodied in consumption increased from 1997 to 2007[23]. Taking four municipalities as examples, almost 50% of the emissions in *Chongqing* were imported from other provinces in 2007, and the proportions were much higher (almost 70%) for *Beijing* and *Shanghai*. Considering the expediting of China’s regional economies, the emissions embodied in provincial trade (EEPT) will definitely be aggravated [24].

Along with the fourth industrial transfer tidal wave, the rigid environmental regulation would inevitably transfer the energy-intensive industries in eastern China to central or western China, which makes exploring the EEPT among the 30 provinces and municipalities (30 provinces hereafter.) of China especially important [25]. Notably, the aforementioned studies primarily focused on EET in China’s 30 provinces in only one year, leaving unresolved the drivers of EEPT, which is used to ensure stakeholders in formulating future climate and environmental policies [13, 26–28]. These topics are what we discuss in this article.

To quantify factors affecting EEPT, a structure decomposition analysis (SDA) based on the multiregional input-output (MRIO) table of China is performed here. To our knowledge, this method has not been used for drivers of emissions embodied in trade at the regional scale before. However, there has been some research using SDA at the national scale, such as emissions embodied in Chinese exports [10, 29], EET among a group of countries[30], and carbon emissions [26, 31–35]. At the regional scale, SDA has only been applied to the CO_2_ emissions of China [36] and to carbon footprints in 8 regions of China [37]. Based on the environmental input-output analysis, SDA is an effective method to analyze the socioeconomic drivers of emissions. Regarding EET on the national scale, Du et al. explored the drivers of emissions embodied in China’s exports to the US during 2002–2007, and the results showed that total export volume was the largest factor in EET between China and the US, followed by intermediate input structure [10]. Regarding EET among different countries, based on the global MRIO table, Xu and Dietzenbacher distinguished the drivers between the local and abroad, and the results showed that the changes in trade structure are distinguishing feature in explaining the uneven growth of EET between developed countries and emerging countries[30]. Until now, the drivers of EEPT have remained unclear, which is not a good condition for China’s reduction. In this context, we analyze the drivers of China’s EEPT, attempting to be favorable to stakeholders in formulating effective mitigation strategies.

The remainder of this study proceeds as follows. Section 2 discuss the methods and data preparation. Section 3 present the results and discussion, with concluding remarks provided in Section 4.

## Methods and data

### Methods

#### Estimating emissions embodied in trade

This study is based on China’s multi-regional IO table. Following works by Serrano and Dietzenbacher (2010) [4], and Xu and Dietzenbacher(2010)[30] (the details are listed in S1 File), EEPT from province *r* to province *s* can be described as

$$\begin{array}{cccc} EEPT_{r-s} & = & \underset{I}{\underset{︸}{\left[\sum_{k=1}^{N}{\left(v^{kr}\right)}^{\prime }\right]F^{rs}}}+\underset{II}{\underset{︸}{{\left(v^{rs}\right)}^{\prime }(\sum_{k=1}^{N}F^{sk})}}, & s\neq r \end{array}$$ (1)

Part *I* of (Eq 1) is EEPT of final consumption. $\sum_{k=1}^{N}v^{kr}$ denotes the carbon emissions to produce one unit of final consumption in province *r*. *F*^*rs*^ represents the consumption of province *s* from province *r*. Part *II* in (Eq 1) is EEPT of intermediate inputs. *v*^*rs*^ denotes intermediate inputs from province *r* for every one unit of final production in province *s*. $\sum_{k=1}^{N}F^{sk}$ represents the total final consumption of province *s*.

Based on the above, emissions embodied in interprovincial outflow (EEPE) and emissions embodied in interprovincial inflow (EEPI) can be expressed as

$$\begin{array}{llllll} EEPE_{r} & = & \sum_{s\neq r}^{N}EEPT_{r-s} & = & \left[\sum_{k=1}^{N}{\left(v^{kr}\right)}^{\prime }\right](\sum_{s\neq r}^{N}F^{rs})+\sum_{s\neq r}^{N}\left[{\left(v^{rs}\right)}^{\prime }(\sum_{k=1}^{N}F^{rk}\right)], & N=30 \end{array}$$ (2)

$$\begin{array}{llllll} EEPI_{r} & = & \sum_{s\neq r}^{N}EEPT_{s-r} & = & \sum_{s\neq r}^{N}\left[\sum_{k=1}^{N}{\left(v^{ks}\right)}^{\prime }\right]F^{sr}+[\sum_{s\neq r}^{N}{\left(v^{sr}\right)}^{\prime }](\sum_{k=1}^{N}F^{rk}), & N=30 \end{array}$$ (3)

Based on the analysis above, the balance of emissions embodied in provincial trade ($E_{r\_p}^{BEET}$) can be expressed as

$$\begin{array}{ccc} E_{r\_p}^{BEET} & = & EEPE_{r}-EEPI_{r} \end{array}$$ (4)

#### Structural decomposition analysis

According to Eqs (2) and (3), EEPE and EEPI are decided by matrices *v* and *F*. Matrix *v* depends on carbon emission intensity *f* and the Leontief inverse matrix *M*, which depends on the direct input coefficient matrix *A* calculated by the structure of intermediate *L* and technology *T*. Matrix *F* depends on the structure of final consumption *S* and the demand matrix of final consumption *q*. We divide each factor into two types to distinguish different effects between the local and the abroad. For instance, *f*^(*r*)^ represents the effect of local carbon emission intensity, while *f*^(−*r*)^ represents the effect of ROC’s (the remainder of China without province *r*) carbon emissions intensity. Detailed factors are listed in Table 1. The relationships of EEPE and EEPI with these factors can be expressed as

$$\begin{array}{lll} EEPE^{r} & = & g^{r}(f^{\left(r\right)},f^{\left(-r\right)},L^{\left(r\right)},L^{\left(-r\right)},T^{\left(r\right)},T^{\left(-r\right)},S^{\left(r\right)},S^{\left(-r\right)},q^{\left(r\right)},q^{\left(-r\right)})\\ EEPI^{r} & = & u^{r}(f^{\left(r\right)},f^{\left(-r\right)},L^{\left(r\right)},L^{\left(-r\right)},T^{\left(r\right)},T^{\left(-r\right)},S^{\left(r\right)},S^{\left(-r\right)},q^{\left(r\right)},q^{\left(-r\right)}) \end{array}$$ (5)

**Table 1:** Description of structural decomposition components of EEPT.

Factors	Description	Factors	Description
*f*^(*r*)^	Emission intensity in province *r*	*f*^(−*r*)^	Emission intensity in ROC
*L*^(*r*)^	Structure of intermediate products in province *r*	*L*^(−*r*)^	Structure of intermediate products in ROC
*T*^(*r*)^	Production technology in province *r*	*T*^(−*r*)^	Production technology in ROC
*S*^(*r*)^	Trade structure of final products in province *r*	*S*^(−*r*)^	Trade structure of final products in ROC
*q*^(*r*)^	Total final demand in province *r*	*q*^(−*r*)^	Total final demand in ROC

In the literature regarding SDA, it is important to note that, when there are many determination factors (*n* ≥ 5), the number of all decomposition forms is n! and different procedures can lead to different results [38–41]. Since we have n = 10, the number of alternatives will be 3,628,800. However, according to the research conducted by Dietzenbacher and Los (1998), the average of two polar decomposition forms can approximate the results of all possible decomposition forms. At the same time, several studies [30, 39, 42] have shown that method of two polar decompositions provides a good approximation of the overall results. Therefore, the two polar decomposition forms are also applied here, and they can be depicted briefly as follows. Decomposition is started by changing the first variable first, followed by changing the second and third variables, etc. to derive the first polar form. The second polar form can be derived in the opposite manner. In other words, decomposition starts by changing the last variable first, followed by changing the second-to-last variable and so forth. Based on the above decomposition method, after calculating the geometric average of $g_{polar1}^{r}$ and $g_{polar2}^{r}$, the change in EEPE from *t* year to *t*-1 year can be obtained, and when the same procedure was performed on EEPI, the equations can be briefly expressed as:

$$\begin{array}{lllll} \Delta EEPE_{t-1,t}^{r} & = & \frac{\Delta EEPE_{t}^{r}}{\Delta EEPE_{t-1}^{r}} & = & \sqrt{g_{polar1}^{r}\times g_{polar2}^{r}}\\ \Delta EEPI_{t-1,t}^{r} & = & \frac{\Delta EEPI_{t}^{r}}{\Delta EEPI_{t-1}^{r}} & = & \sqrt{u_{polar1}^{r}\times u_{polar2}^{r}} \end{array}$$ (6)

### Data

In this study, we use data from 2002, 2007 and 2010. The MRIO tables for China in 2002 covering the 8 sectors used in this paper were constructed and compiled by Wang and colleagues [43]. The MRIO tables in 2007 and 2010 covering 30 sectors for China came from the study conducted by Liu and colleagues [44, 45]. To remove deflation effects, our study treats economic data at constant prices, so the MRIO tables of 2007 and 2010 were maintained at 2002 constant prices. The price deflators from the statistical yearbook were applied. Due to a lack of data from Tibet, Hong Kong, Taiwan and Macao, the research subjects are 30 provinces in China without these four provinces.

The sectors in the MRIO tables and those in energy balance tables are not the same: the first has 30 sectors, and the second has 6 sectors. There are two methods to render sectors consistent: one is to split the energy balance table according to the IO table, and the other is the opposite. Each of these approaches has advantages and disadvantages. The first approach can guarantee the data integrity of the IO table, and the second one can avoid errors caused by adding extra energy consumption [46]. Based on the data we have, the second approach is applied here. We use sectors in the energy balance table and combine sectors in the IO table. The detailed sectors are farming, forestry, animal husbandry and fishery, industry, construction, transport, storage and post, wholesale, retail trade and hotel, restaurants, and other services. The carbon emissions are calculated using the energy balance tables of each province. The CO_2_ emissions factors of various types of energy come from the Intergovernmental Panel on Climate Change (IPCC) reference approach and Guan et al.(2012)[47]. The sectoral carbon intensity is the ratio of sectoral emissions to sectoral output.

## Results and discussion

### Drivers of emissions embodied in provincial trade

First, we consider the total emissions embodied in provincial trade (EEPT) of China, which is the summation of all provinces’ EEPE (EEPI). The ratio between EEPT and production-based emissions increases from 35.05% (1346.39 Tg) in 2002 to 40.24% (3004.08 Tg) in 2010. The increasing ratio between EEPT and production-based emissions indicates two things: one is that the economic crisis did not have a negative effect on EEPT, and the other is that there are carbon emissions transfers among provinces, along with accelerated interprovincial trade. The question is: What are factors of EEPT?

Tables 2 and 3 show the decomposition analysis results of EEPE and EEPI, respectively. The first column is the proportion of changes in EEPE (EEPI), followed by the effects generated by changes in carbon intensity (Δ*f*^(*r*)^, Δ*f*^(−r)^), changes in the structure of intermediate inputs (Δ*L*^(r)^, Δ*L*^(−r)^), changes in technology (Δ*T*^(r)^, Δ*T*^(−r)^), changes in the trading structure (Δ*S*^(r)^, Δ*S*^(−r)^) and changes in the final demand (Δ*q*^(r)^, Δ*q*^(−r)^).

**Table 2:** Structure decomposition analysis of EEPE (2002–2010) (unit: %).

	Province	Δ*EEPE*	Δ*f*^(r)^	Δ*f*^(−r)^	Δ*L*^(r)^	Δ*L*^(−r)^	Δ*T*^(r)^	Δ*T*^(−r)^	Δ*S*^(r)^	Δ*S*^(−r)^	Δ*q*^(r)^	Δ*q*^(−r)^
Eastern	Beijing	91.03	-26.12	-1.15	-29.52	2.00	13.78	0.57	5.70	0.71	0.09	198.44
	Tianjin	252.57	-10.16	-1.01	9.86	0.23	1.44	4.25	17.81	0.53	0.95	184.80
	Hebei	165.20	-4.23	-0.80	-5.61	-0.01	3.73	7.39	-9.42	0.46	1.36	187.90
	Shanghai	378.13	-9.40	-1.51	-14.06	0.49	17.70	-2.01	75.84	2.37	0.46	197.43
	Jiangsu	515.48	42.16	-1.67	2.36	-0.28	0.61	7.60	30.13	1.75	0.91	198.22
	Zhejiang	190.73	37.53	-1.23	-27.88	-0.18	-3.72	8.49	-2.97	1.23	0.41	188.57
	Fujian	508.83	58.99	-0.67	-6.15	0.07	6.35	10.19	20.50	0.59	0.37	187.96
	Shandong	377.04	20.65	-0.79	-12.55	0.08	14.86	5.10	24.55	1.55	0.48	196.82
	Guangdong	460.75	19.29	-1.55	-0.62	0.77	14.70	4.87	30.88	2.06	1.03	193.70
	Hainan	25.61	-40.43	-0.60	-14.59	1.12	-5.38	3.40	-13.35	-0.41	0.04	190.79
Central	Shanxi	246.34	-33.63	-0.03	68.77	0.79	-0.15	13.68	-4.52	-2.27	1.21	186.25
	Anhui	90.58	-44.38	0.34	-21.78	-0.80	6.59	4.85	33.01	1.28	0.28	191.52
	Jiangxi	201.82	-36.31	-0.31	31.53	-0.66	10.70	10.00	4.32	0.96	0.32	182.77
	Henan	193.96	-5.55	-0.35	14.60	0.26	-41.52	12.51	38.48	1.15	1.39	190.93
	Hubei	96.85	-23.74	-0.26	-22.00	0.38	-0.11	7.36	5.81	0.41	0.25	189.39
	Hunan	214.54	-21.12	-0.28	-18.77	0.02	8.42	8.61	40.88	0.88	0.46	192.75
Western	Guangxi	96.46	-11.14	-0.68	-20.96	0.57	0.22	9.66	-9.23	0.17	1.06	177.29
	Inner Mongolia	632.14	-10.74	0.00	117.87	0.30	7.89	7.93	12.41	-0.16	1.68	182.44
	Chongqing	47.62	-41.81	-0.50	-0.12	0.84	0.97	4.65	-20.52	-0.76	0.20	203.11
	Sichuan	248.80	-27.21	-0.24	5.81	1.99	-1.37	6.97	44.20	0.08	0.39	191.20
	Guizhou	226.47	-9.30	-0.24	-5.69	0.64	6.31	8.45	12.56	0.17	0.47	191.11
	Yunnan	292.44	28.28	-0.58	-20.22	0.08	-0.65	12.34	20.35	-0.06	1.01	184.22
	Shaanxi	386.12	2.01	-0.71	27.51	0.48	-6.50	4.52	28.68	0.41	1.10	193.46
	Gansu	284.69	23.93	0.64	12.04	0.29	-9.16	7.96	-3.06	-0.48	0.15	189.65
	Qinghai	127.22	-28.84	0.02	62.30	4.63	3.11	3.99	-38.94	-1.19	0.32	189.63
	Ningxia	284.32	19.27	-0.13	4.50	3.01	3.50	3.96	-4.18	-1.27	0.22	193.82
	Xinjiang	317.88	51.56	-0.08	-2.13	1.96	-3.22	2.53	-1.10	-0.20	0.38	181.28
Northeastern	Liaoning	269.62	17.88	0.36	-17.35	0.06	-2.07	7.96	15.82	0.83	0.81	203.54
	Jilin	200.37	10.59	0.18	-14.38	-0.13	1.88	6.35	-0.03	0.41	1.37	187.57
	Heilongjiang	308.89	38.78	0.20	-18.46	-1.63	3.83	5.68	12.13	0.55	1.17	192.86

**Table 3:** Structure decomposition analysis of EEPI (2002–2010) (%).

	Province	Δ*EEPE*	Δ*f*^(r)^	Δ*f*^(−r)^	Δ*L*^(r)^	Δ*L*^(−r)^	Δ*T*^(r)^	Δ*T*^(−r)^	Δ*S*^(r)^	Δ*S*^(−r)^	Δ*q*^(r)^	Δ*q*^(−r)^
Eastern	Beijing	152.60	-0.07	-4.03	-3.20	5.76	16.51	2.68	0.62	0.15	73.31	23.12
	Tianjin	422.33	-0.04	-7.67	0.32	5.12	3.75	3.04	23.91	0.08	242.03	18.36
	Hebei	196.14	-0.06	-7.46	-3.89	4.02	3.39	-1.31	6.82	-0.02	146.29	19.33
	Shanghai	507.11	-0.03	-5.90	24.07	4.41	20.12	-1.17	58.44	0.45	126.06	16.65
	Jiangsu	359.26	0.19	-9.36	36.95	4.79	5.65	-0.80	20.56	0.22	146.20	13.04
	Zhejiang	160.21	0.11	-5.87	4.25	4.67	-1.98	-0.18	-2.98	0.11	120.58	20.72
	Fujian	175.37	0.10	-6.74	-3.10	4.76	7.06	1.57	20.20	0.02	110.34	5.68
	Shandong	117.86	0.10	-7.47	-27.18	5.78	21.99	-0.36	-10.87	0.27	153.55	10.87
	Guangdong	323.02	0.08	-6.19	3.93	4.66	28.43	-0.05	22.41	0.34	135.23	11.68
	Hainan	-25.61	-0.04	-6.07	-49.93	4.24	-4.06	2.92	-28.73	-0.01	95.75	10.21
Central	Shanxi	546.12	-0.62	-2.98	0.80	4.70	2.45	0.97	65.10	0.02	251.43	5.79
	Anhui	168.58	-0.37	2.18	-17.19	5.71	14.62	-0.59	-1.43	0.27	120.38	21.43
	Jiangxi	71.24	-0.13	-6.64	-40.43	4.79	10.44	0.57	-4.73	0.01	158.06	7.74
	Henan	259.88	-0.06	-5.64	0.06	5.89	-28.07	6.23	32.68	0.17	215.87	12.28
	Hubei	82.95	-0.08	-6.15	-22.11	4.46	3.90	-2.53	-9.79	0.02	146.74	6.35
	Hunan	222.29	-0.10	-3.52	-21.79	4.95	18.00	-1.00	10.09	0.14	184.95	11.02
Western	Guangxi	233.87	-0.06	-11.14	-22.63	4.44	1.23	4.42	0.92	0.00	288.26	12.32
	Inner Mongolia	641.38	-0.18	-1.30	-17.13	4.46	7.65	0.00	37.74	0.03	447.75	7.01
	Chongqing	30.57	-0.27	-7.31	-31.11	4.52	2.50	1.29	-4.45	-0.53	57.93	25.85
	Sichuan	180.90	-0.09	-4.42	-21.80	5.47	8.43	-1.02	15.44	0.07	170.60	6.32
	Guizhou	215.28	-0.03	-10.49	-10.77	4.41	6.07	3.14	21.19	0.01	161.79	8.93
	Yunnan	294.35	0.10	-10.78	0.16	5.42	-1.66	3.72	20.06	0.03	219.24	6.93
	Shaanxi	342.05	0.01	-7.27	-0.82	5.64	-2.42	-3.99	33.97	0.13	217.83	13.91
	Gansu	175.79	0.04	2.16	-0.04	5.51	-3.06	-2.37	1.36	0.01	138.29	11.92
	Qinghai	122.73	-0.06	-3.87	-24.86	4.74	3.48	1.43	-2.84	-0.06	146.31	17.34
	Ningxia	207.07	0.05	-7.48	-14.75	5.24	8.53	-0.87	2.61	0.00	208.05	8.74
	Xinjiang	202.92	0.14	-3.16	-3.41	5.66	8.48	-5.97	8.44	0.03	158.03	7.19
Northeastern	Liaoning	323.87	0.12	3.44	7.92	5.16	3.89	2.37	11.22	0.33	163.00	15.55
	Jilin	255.70	0.10	-0.53	-8.30	4.90	1.94	2.05	0.11	0.24	197.13	19.71
	Heilongjiang	309.88	0.32	0.51	-0.43	5.79	7.24	2.22	40.75	0.07	128.26	9.49

Taking *Shanghai* as an example, with all of the other factors remaining constant, changes in local carbon intensity (Δ*f*^(r)^) can only decrease EEPE by 9.40%. In other words, the quantitative relationship between the changes in EEPE (Δ*EEPE*) and the changes in these driving factors is Δ*EEPE* + 1 = (Δ*f*^(r)^ + 1) × (Δ*f*^(−r)^ + 1) × (Δ*L*^(r)^ + 1)…× (Δ*q*^(−r)^ + 1). For *Shanghai*, the relationship is 4.78 = 0.91×0.99×0.86×1.00×1.18×0.98×1.76×1.02×1.00×2.97.

From 2002 to 2010, the EEPE of all 30 of these provinces increased. *Inner Mongolia* had the greatest rate of increase, and it increased 6.32 times over that in 2002. The other provinces with large increasing rates were *Jiangsu* (5.15), *Fujian* (5.09) and *Guangdong* (4.61). Regarding EEPI, *Inner Mongolia* also had the greatest rate of increase, and it increased 6.41 times over that in 2002, followed in decreasing order by *Shanxi* (5.46), *Shanghai* (5.07) and *Tianjin* (4.22).

From Table 2, we find that the growth of EEPE is concentrated in a group of provinces, and the top 10 provinces with increasing EEPE contribute almost 60.45% of the changes in total EEPE. Among these changes, the increase in EEPE in *Hebei* constitutes the largest contribution, with a proportion of 8.83%, also providing information about the situation in which the largest proportion of total EEPE is always in *Hebei*. Increases in the following 9 provinces constitute 51.63% of the total increase.

According to Tables 2 and 3, changes in final demand would lead to a greater increase in EEPT. For instance, on average, changes in local final demand (Δ*q*^(r)^) would cause EEPI to increase by 170.98%. Carbon intensity (Δ*f*^(r)^, Δ*f*^(−r)^) did not have an obvious effect on EEPT on average, while the effects changed between different provinces. In Table 2, local carbon intensity (Δ*f*^(r)^) has an obvious effect on EEPE. For most provinces, local carbon intensity (Δ*f*^(r)^) has a negative effect on EEPE, while in *Zhejiang* and *Jiangsu*, the positive effect is approximately 40.00%. One possible reason for this outcome is that the carbon intensity of these two provinces in 2010 increased compared with that in 2002, especially in the industries of transport, storage and post. There are other provinces in which the local carbon intensity (Δ*f*^(r)^) also has a positive effect on EEPE, such as in *Fujian*, *Yunnan* and *Shaanxi*, possibly because, although the carbon intensity of these provinces decreased as a whole, with regard to sectoral carbon intensity, the carbon intensity of industry and transport, storage and post increased. The effect of changes on technology (Δ*T*^(r)^, Δ*T*^(−r)^) is very small, 1.76–6.66% on average. It is notable that technology in resource-heavy provinces, such as *Hebei*, *Shanxi*, and the western provinces, did not change very much, demonstrating that they could be improved in the future. Here, we found that, for northeastern provinces, regardless of the changes in local technology or the changes in the other provinces’ technology, EEPE would decrease, indicating that the traditional industrial base of China improved technology from 2002 to 2010, which is good for the carbon emissions of all of China.

The effect of Δ*q*^(r)^(Δ*f*^(r)^) on EEPE (EEPI) is almost 0, except for only a few provinces (*Shanxi*, *Inner Mongolia* and *Jilin*) with a small effect of 0.50–1.50%. These effects are related to $\sum_{\text{s}\neq r}v^{rs}f^{sr}$ in Eqs (2) and (3), indicating the emissions embodied in the other provinces’ final consumption of region *r*. These parts of the products are exported to the other provinces as intermediate inputs from region *r* and then are imported to region *r* as final consumption, which is also why this part of emissions exists in both equations. Generally, the other provinces’ demands and local production structures affect EEPE mostly, while local demand and the other provinces’ production structures affect EEPI mostly. However, assuming that the other provinces’ carbon intensity (the other provinces’ final consumption demands) has nothing with EEPE (EEPI) is definitely wrong because there are indirect connections within them. For example, there are emissions contained in intermediate inputs, which are imported from other provinces to satisfy the production of local final demand.

From Tables 2 and 3, the effect of local carbon intensity (Δ*f*^(r)^) on EEPE differs greatly among provinces, from -44.38% to 58.99%, while the effect of the other provinces’ carbon intensity (Δ*f*^(−r)^) on EEPI varies slightly, from -11.14% to 3.44%. The effect of the other provinces’ final demand (Δ*q*^(−r)^) on EEPE varies slightly, from 177.29% to 203.54%, while the effect of local carbon intensity (Δ*q*^(r)^) on EEPI differs greatly among the provinces, from 57.93% to 447.75%. This finding illustrates that, for any provinces *s* and *r*, if they have differences in carbon intensity, then they would exist in local carbon intensity (Δ*f*^(r)^ and Δ*f*^(s)^) and not in the other provinces’ carbon intensity (Δ*f*^(−r)^ and Δ*f*^(−s)^) because both changes of Δ*f*^(−s)^ and Δ*f*^(−r)^ contain overlapped effects of the other 28 provinces (except for provinces *s* and *r*).

From Table 3, changes in local product technology (Δ*T*^(r)^) would cause an increase in EEPI of 5.82%, indicating that each product in 2010 contains more intermediate input and less value than in 2002. This result is consistent with the research on the whole world from 1995 to 2007[30], which found that an improvement in product technology would cause a 15.00% increase in EET. On the sectoral scale, except for wholesale, retail trade and hotel, restaurants, and other services, the other sectors all had higher proportions of intermediate inputs in 2010 than in 2002, indicating that each final product needs more intermediate inputs, resulting in more carbon emissions.

Along with international product activities transferred from developed countries to undeveloped countries, the emissions are transferred as well, and for the lower increasing rate of EEI and the higher increasing rate in developed countries, the EET is increased [30]. This phenomenon occurs in China’s provinces as well. From 2002 to 2010, the developed eastern provinces, such as *Beijing* and *Shanghai*, had higher increasing rates of EEPI and a lower growing rate of EEPE; even their EEPI was already much higher than their EEPE, resulting in the net balance of EEPT ($E_{r\_p}^{BEET}$) of these provinces increasing over time. It should be noted that the reduction policy of energy resource-rich provinces has begun to take effect positively. Taking *Shanxi* as an example, although the increasing rate on EEPE is high, it also has a high increasing rate on EEPI, indicating that *Shanxi* is distributing its reduction pressure. Regarding western resource-rich provinces, such as *Guizhou*, *Gansu*, *Qinghai*, *Ningxia*, and *Xinjiang*, they have a lower increasing rate on EEPI and a higher increasing rate on EEPE; in other words, reduction pressure continues to grow.

From the perspective of the contribution of absolute increases in EEPI and EEPE on total EEPT, the central provinces, such as *Shanxi*, and *Henan*, have become increasingly important in Chinese provincial trade. Briefly, the central provinces contribute 16.76% and 19.94% of the increase in EEPI and EEPE, respectively. For each single province, increasing rate of EEPI (EEPE) is 200.00% on average, while the net increase in EEPI remains larger than that of EEPE. Therefore, $E_{r\_p}^{BEET}$ of the central provinces continues to increase, and the reduction pressure cannot be overstated.

Changes in trade structure in the other provinces’ final demand (Δ*S*^(−r)^) would cause EEPE to increase by 0.39% on average. It is well known that *Shanxi* is an important coal resource base, and it should be mentioned that Δ*S*^(−r)^ of *Shanxi* causes its EEPE to decrease by 2.27%, indicating that, under the reduction policy, China’s provinces are adjusting their industrial structures to reduce the consumption of coal, resulting in emissions reductions in *Shanxi*. For coastal provinces, such as *Shanghai*, *Zhejiang* and *Jiangsu*, the effects of Δ*S*^(−r)^ on EEPE are positive and obvious. Changes in the trade structure of local final demand (Δ*S*^(r)^) would cause EEPI to increase by 12.96% on average, and the positive effect usually occurs in the eastern provinces; for example, changes in Δ*S*^(r)^ cause EEPI in *Shanghai* to increase by 58.44%. Changes in the trade structure of the other provinces’ final demand (Δ*S*^(−r)^) would cause EEPI to increase by 0.09% on average. Regarding central provinces, such as *Henan* and *Hunan*, the effect of Δ*S*^(−r)^ is relatively larger, almost 2–3 times the average value. It should be noted that the effect of Δ*S*^(r)^ on EEPI is much larger than the effect of Δ*S*^(−r)^. The reason for this outcome is that the effect of Δ*S*^(r)^ on EEPI is indirect. Taking *Henan* as an example, if the other provinces need more production from *Henan*, it would cause *Henan* to import more intermediate inputs for final production (EEPI of *Henan* growing 0.17%).

Similar to the trade structure of final consumption, changes in the structure of local intermediate input (Δ*L*^(r)^) would cause EEPE to increase by 2.81%, while changes in structure in the other provinces’ intermediate inputs (Δ*L*^(−r)^) would cause EEPE to increase by 0.58%. This phenomenon is likely to be obvious in western provinces, such as *Qinghai*, *Ningxia*, and *Xinjiang*, the EEPE of which would increase by 1.96–4.63% due to changes of structure, compared to the other provinces’ intermediate input (Δ*L*^(−r)^), indicating that western provinces are taking positions in the production processes of intermediate input. Therefore, an old industrial base such as *Heilongjiang* or *Jilin* lost its economic position to some degree. Δ*L*^(−r)^ will cause its EEPE to decrease by 0.13%-1.63%. Regarding EEPI, Δ*L*^(r)^ would cause it to decrease by 8.88% on average, indicating that the adjustment of the industrial structure in China is useful.

### Specific provinces

In this subsection, we discuss the EEPT results of two specific provinces (*Beijing* and *Shanxi*) in detail. *Beijing* is the capital of China, with a higher EEPI and lower EEPE. *Shanxi* is a typical resource-rich province in central China, with a higher EEPE and lower EEPI.

#### Beijing

The EEPE in *Beijing* grew by 91.03% (28.06 Tg) from 2002 to 2010, which was far less than the average rate of China (257.75%). Compared with other provinces, this difference was not caused by lower growth in final demand compared to other provinces but by a larger reduction in *Beijing*’s emission intensities. The changes in final demand of the other provinces led to a 198.44% increase in EEPE, which was quite close to the average (190.65%). Changes in *Beijing*’s emission intensities led to a 26.12% reduction in EEPE, which was larger than the average reduction (0.44%). Important factors that reduced *Beijing*’s EEPE were changes in the structure of intermediate inputs in *Beijing* (causing a 29.52% reduction) and changes in the technology in *Beijing* (causing a 29.52% increase). The increasing effects of changes in the technology in *Beijing* indicated that the average output in *Beijing* contained less domestic value added than earlier. These two factors also differed greatly from the average effect (causing 2.81% and 1.76% growth, respectively).

The EEPI in *Beijing* increased by 152.60% (82.19 Tg) from 2002 to 2010, which was less than the average rate in China (242.52%). Important factors include changes in final demand in *Beijing* (causing 73.31% growth) and emission intensity in other provinces (causing a 4.03% decrease). The effect of emission intensity in other provinces is quite close to the average level (causing a 5.04% decrease). The effect of changes in final demand is much smaller than the average effect (causing 170.97% growth). Another change with a large positive effect is technology in *Beijing*, causing 16.51% growth, which is much larger than the average (causing 5.82% growth). This finding indicates that the technology in *Beijing* is not produced very cleanly and would still need to be improved.

The changes in trade structure of intermediate input (both in *Beijing* and other provinces) caused *Beijing*’s EEPE to decrease by 28.11% and *Beijing*’s EEPI to increase by 2.38%, while the changes in trade structure of final demand (both in *Beijing* and other provinces) caused *Beijing*’s EEPE to increase by 6.45% and *Beijing*’s EEPI to remain stable (increasing by 0.77%).

#### Shanxi

*Shanxi* is a most important energy-rich province in China with rich coal resources. The EEPE of *Shanxi* increased by 246.34% from 2002 to 2010. The changes in other provinces’ final demand played a very important role in the increase in emissions embodied in provincial exports, accounting for an increase of 186.25%. The next largest positive effect were changes in *Shanxi*’s structure of intermediate inputs, accounting for 68.77% growth, which was much larger than the average (causing 2.81% growth). The emission intensities in *Shanxi* caused a 33.63% deduction, which was much larger than the average (0.44%). The next most negative effect were changes in other provinces’ trade structure of final demand, inducing a decrease of 4.52%, which indicated that the other provinces changed their structures to reduce their use of energy production in *Shanxi*, resulting in a decrease in *Shanxi*’s EEPE. However, despite the larger than average reduction in *Shanxi* due to its lower emission intensity, the increased reliance of other provinces on imports from *Shanxi* led to a large increase in EEPE.

The EEPI of *Shanxi* increased by 546.12% from 2002 to 2010, which was much more than its EEPE. The most important factor was the change in final demand of *Shanxi*, causing 251.43% growth, which was larger than the average (causing 170.97% growth). The next largest positive effect were changes in *Shanxi*’s trade structure of final demand, inducing an increase of 65.10%. The changes in other provinces’ structures of intermediate input caused *Shanxi*’s EEPI to increase by 4.70%.

The changes in *Shanxi*’s structure of intermediate input caused *Shanxi*’s EEPI to increase by 0.80%, and it caused *Shanxi*’s EEPE to increase by 68.77%. This involvement in processing trade indicated that *Shanxi* became more dependent on imported intermediate inputs. In 2002, 84.46% of *Shanxi*’s intermediate input was produced at home, and 15.64% of the intermediate input was produced by other provinces. The intermediate input produced at home decreased by 79.19% in 2010.

The changes in trade structure of intermediate input (both in *Shanxi* and other provinces) caused *Shanxi*’s EEPE to increase by 70.10% and *Shanxi*’s EEPI to increase 5.54%. At the same time, the changes in trade structure of final demand (both in *Shanxi* and other provinces) caused *Shanxi*’s EEPE to decrease by 6.69% and *Shanxi*’s EEPI to increase by 65.13%. First, this outcome indicates that, as an energy-rich province, *Shanxi* still plays an important role in the production of intermediate inputs. Second, *Shanxi* is attempting to reduce its emissions by importing final products from other lower emission intensity provinces. The share of final demand produced at home decreased from 93.21% (in 2002) to 84.21% (in 2010).

## Conclusions and policy implications

Based on MRIO tables from 2002, 2007 and 2010, we calculated emissions embodied in provincial trade (EEPT) in 30 of China’s provinces. The results showed that China’s EEPT increased from 2002 to 2010, constituting approximately 40% of the emissions based on production. That indicates the important role of EEPT on the reduction strategies. To determine the driving forces of EEPT, structure decomposition analysis was used here. The decomposition results showed that final demand and carbon intensity were key factors for EEPT. Provinces should adopt different strategies to reduce the effects of EEPT, which is related to the reduction responsibilities based on different calculation methods (the production-based emissions or the consumption-based emissions). On one hand, from the consumption perspective, local final demand and abroad carbon intensity had crucial effects on EEPI, that means decreasing local final demand from other provinces or importing productions from provinces with low carbon intensity are best ways for the mitigation on EEPI. On the other hand, from the production perspective, the other provinces’ final demand and local carbon intensity affected EEPE significantly, that means limiting the interprovincial export of energy-commodities or reducing local carbon emission intensity are useful for reduction on EEPE. However, these changes among provinces are comparable, so they are not the key points for interpreting the inequality between the developed eastern provinces and the western and central provinces.

The trade structure is the distinguishing feature in explaining the uneven growth of EEPT among the eastern, western, central, and northeastern provinces. In terms of intermediate inputs, producers are more likely than developed provinces to import products from undeveloped western provinces or northeastern provinces or to use local products. In *Jiangsu* and *Zhejiang*, for example, the changes in the other provinces’ trade structure of intermediate inputs led to decreases in EEPE of 0.28% and 0.18%, respectively, while among the western provinces, the changes in the other provinces’ trade structure of intermediate inputs caused EEPE to increase by 1.34% on average. Regarding final products, consumers were inclined to import consumption from developed eastern provinces rather than from central and western provinces. On average, a 0.39% increase in EEPE was generated by the trading structure of the other provinces’ final consumption. With regard to developed provinces, such as *Shanghai*, *Guangdong*, and *Jiangsu*, this increasing rate could be as high as 2.37% while in the central, western, and northeastern provinces, this rate was generally small and even negative in some regions.

Another main finding of this research is that the developed eastern provinces did not act in the same manner regarding China’s production in 2010 as they did in 2002, and the western and central provinces began to participate more in production processes in China. Consequently, the consumption-based carbon emissions (related to EEPI) of eastern provinces increased obviously, while their production-based emissions (related to EEPE) did not change too much. For example, the ratio of interprovincial exports of intermediate inputs in *Zhejiang* to total interprovincial export decreased by 0.86% in 2010 compared to 2002, while the ratio of interprovincial exports of final consumption to total interprovincial exports decreased by 3.22%. For central provinces, the ratio of the interprovincial exports of intermediate inputs in *Inner Mongolia* to the total interprovincial export increased by 0.27% in 2010 over 2002, while the ratio between the interprovincial exports of final consumption and the total interprovincial export increased by 3.31%. Regarding the western provinces, these two ratios were, respectively, increased by 2.69% and decreased by 3.84%.

Considering the increasing participation of western and central provinces in the production chain, how to reduce their EEPE without reducing the interprovincial export is quite crucial. One possible way is to adjust their local intermediate and trading structure to low energy intensity structure by levying a carbon tax on energy industries or subsidies on new energy industries, like *Shanxi*, *Qinghai Henan*, *Hunan* and *Inner Mongolia*, whose local intermediate or trading structure has high positive effects on their EEPE. About the technology in production, most provinces’ technology has improved and has a negative effect on their EEPE, while there still are some provinces’ technology need to be improved, such as *Anhui*, *Jiangxi* and *Inner Mongolia*. We should notice that the other regions’ technology has positive effects on EEPE of the western and central provinces. That means the technology improved in whole China is necessary due to the production corporation among provinces.

As for eastern provinces, such as *Beijing*, *Shanghai*, *Guangdong*, and *Jiangsu*, it’s necessary to reduce the EEPI while satisfying their production need at the same time. One possible way is improving the other regions’ technology, which has a negative effect on their EEPI. Considering the local trade structure of the intermediate and the final demand, there are some positive effects in *Shanghai*, *Jiangsu* and *Guangdong*, this indicates the cleaner materials and the less carbon-intensity productions should be encouraged during both the production and consumption processes.

Generally speaking, interprovincial trade resulted in an increase in total EEPT, and the inequalities between EEPT of the eastern provinces and that of the central and western provinces exacerbated carbon emissions transfers. The northeastern, western and central provinces should be on guard not to let the energy-intensive industries of developed eastern provinces in. Meanwhile, so as to realizing the reduction goal as a whole, the eastern provinces with higher EEPI and lower EEPE (like *Beijing*, *Shanghai*), should consider helping the provinces with higher EEPE and lower EEPI (such as *Guizhou*, *Gansu and Qinghai*) through the technology transfer or environmental compensation through regional corporations.

## Supporting information

S1 File Methods for estimating emissions embodied in trade and structural decomposition analysis. (PDF)
