# Supplementary material for: Examining drivers of the emissions embodied in trade
Source: PLoS One. 2017 Apr 20;12(4):e0176159. doi: 10.1371/journal.pone.0176159 (PMC5398700; doi:10.1371/journal.pone.0176159)
Supplement: S1 File — (PDF) [file pone.0176159.s001.pdf]

Methods for estimating emissions embodied in trade and structural decomposition analysis

According to the Chinese multi-regional IO table, the intermediate input matrix can be expressed as:

$$Z_t = \begin{bmatrix} Z_t^{1,1} & \cdots & Z_t^{1,p} & \cdots & Z_t^{1,N} \\ \vdots & \ddots & & & \vdots \\ Z_t^{r,1} & \cdots & Z_t^{r,p} & \cdots & Z_t^{r,N} \\ \vdots & & & \ddots & \vdots \\ Z_t^{N,1} & \cdots & Z_t^{N,p} & \cdots & Z_t^{N,N} \end{bmatrix} \quad (1)$$

where Z_t^{rp} for $r,p=1,2\cdots,N$ represents the matrix with intermediate deliveries $Z_{ij,t}^{rp}$ (while $i,j=1,2\cdots,n$) form sector i in region r to sector j in region p at time t. The intermediate input coefficients are obtained as $a_{ij,t}^{rp} = z_{ij,t}^{rp} / x_{j,t}^{p}$, where $x_{j,t}^{p}$ gives the gross domestic output of sector j in region p at time t. The input Coefficients matrix A_t, which has the same structure with Z_t can be expressed as:

$$A_t = \begin{bmatrix} A_t^{1,1} & \cdots & A_t^{1,p} & \cdots & A_t^{1,N} \\ \vdots & \ddots & & & \vdots \\ A_t^{r,1} & \cdots & A_t^{r,p} & \cdots & A_t^{r,N} \\ \vdots & & & \ddots & \vdots \\ A_t^{N,1} & \cdots & A_t^{N,p} & \cdots & A_t^{N,N} \end{bmatrix} \text{and } X = \begin{bmatrix} x_t^{1} \\ \vdots \\ x_t^{r} \\ \vdots \\ x_t^{N} \end{bmatrix} \quad (2)$$

The final demand matrix for every region can be expressed as:

$$F_t = \begin{bmatrix} f_t^{1,1} & \cdots & f_t^{1,p} & \cdots & f_t^{1,N} \\ \vdots & \ddots & \vdots & & \vdots \\ f_t^{r,1} & \cdots & f_t^{r,p} & \cdots & f_t^{r,N} \\ \vdots & & \vdots & \ddots & \vdots \\ f_t^{N,1} & \cdots & f_t^{N,p} & \cdots & f_t^{N,N} \end{bmatrix} \quad (3)$$

On the basis of the equilibrium between the column and the row, we have

$$x_t^r = \sum_{p=1}^{N} Z_t^{rp} u + \sum_{p=1}^{N} f_t^{rp} u \quad (4)$$

where u is a vector consisting of ones of the appropriate length. For the whole set of regions, we can write $x_t = Z_t u + F_t u$, or $x_t = A_t x_t + F_t u$. So we can get $Z_t u = A_t x_t$ and $x_t = (I - A_t)^{-1} F_t u$, the Leontief inverse matrix can be expressed as $M_t \equiv (I - A_t)^{-1}$.

The full emission coefficients for each region are given as follows:

$$V_t = \begin{bmatrix} (w_t^1)' M_t^{1,1} & \cdots & (w_t^1)' M_t^{1,p} & \cdots & (w_t^1)' M_t^{1,N} \\ \vdots & \ddots & \vdots & & \vdots \\ (w_t^r)' M_t^{r,1} & \cdots & (w_t^r)' M_t^{r,p} & \cdots & (w_t^r)' M_t^{r,N} \\ \vdots & & \vdots & \ddots & \vdots \\ (w_t^N)' M_t^{N,1} & \cdots & (w_t^N)' M_t^{N,p} & \cdots & (w_t^N)' M_t^{N,N} \end{bmatrix} \quad (5)$$

where the direct emission coefficients for region r can be given by $w_{i,t}^r = e_{i,t}^r / x_{i,t}^r$, $e_{i,t}^r$ represents the amount of the CO_2 emissions in sector i of region r. In Equation (5) $(v_t^{rp})' = (w_t^r)' M_t^{rp}$, where $v_{i,t}^{rp}$ give the CO_2 emissions (directly and indirectly) generated in region r for one unit of final demand in region p for good i.

EEPT from province *r* to province *p* can be described as

$$EEPT_{r-p} = \underbrace{\left[\sum_{k=1}^{N} (v^{kr})' \right] F^{rp}}_{I} + \underbrace{(v^{rp})' (\sum_{k=1}^{N} F^{pk})}_{II}, p \neq r \quad (6)$$

Part *I* of equation (1) is EEPT of final consumption. $\sum_{k=1}^{N} v^{kr}$ denotes the carbon emissions to produce one unit of final consumption in province *r*. F^{rp} represents the consumption of province *p* from province *r*. Part *II* in equation (1) is EEPT of intermediate inputs. v^{rp} denotes intermediate inputs from province *r* for every one unit of final production in province *p*. $\sum_{k=1}^{N} F^{pk}$ represents the total final consumption of province *p*.

Based on the above, emissions embodied in interprovincial outflow (EEPE) and emissions embodied in interprovincial inflow (EEPI) can be expressed as:

$$EEPE_r = \sum_{p \neq r}^{N} EEPT_{r-p} = \left[\sum_{k=1}^{N} (v^{kr})' \right] (\sum_{p \neq r}^{N} F^{rp}) + \sum_{p \neq r}^{N} [(v^{rp})' (\sum_{k=1}^{N} F^{rk})], N = 30 \quad (7)$$

$$EEPI_r = \sum_{p\neq r}^{N} EEPT_{p-r} = \sum_{p\neq r}^{N}\left[\sum_{k=1}^{N}(v^{kp})^{'}\right]F^{pr} + [\sum_{p\neq r}^{N}(v^{pr})^{'}](\sum_{k=1}^{N}F^{rk}), N=30 \quad (8)$$

Based on the analysis above, the balance of emissions embodied in provincial trade ($E_{r\_p}^{BEET}$) can be expressed as

$$E_{r\_p}^{BEET} = EEPE_r - EEPI_r \quad (9)$$

As for structural decomposition analysis, the input matrix $A^{r,p}$ in Equation (2) represent the input coefficients between each two regions in Chinese IO table, which also show the origin input. However, during the production, the so-called technological coefficients for region r can be obtained by summing all intermediate input matrices for region r .

$$T_t^r = \sum_{p=1}^{N} A_t^{pr} \quad (10)$$

Given the certain technological input requirements, the trade structure of intermediate input represents the directions of inputs can be described by

$$L_t = \begin{bmatrix} l_t^{1,1} & \cdots & l_t^{1,p} & \cdots & l_t^{1,N} \\ \vdots & \ddots & \vdots & & \vdots \\ l_t^{r,1} & \cdots & l_t^{r,p} & \cdots & l_t^{r,N} \\ \vdots & & \vdots & \ddots & \vdots \\ l_t^{N,1} & \cdots & l_t^{N,p} & \cdots & l_t^{N,N} \end{bmatrix}, \quad l_{ij,t}^{pr} = a_{ij,t}^{pr} \big/ t_{ij,t}^{r} \text{ as } A_t^{pr} = L_t^{pr} \otimes T_t^r$$

(11)

where $\otimes$ means the Hadamard product of element-wise multiplication.

In a similar way, the final demand matrix F can be split into the total final demand and the trade structure of final products. The final demand vector for region r is defined as $q_t^r = \sum_{p=1}^{N} f_t^{pr}$.

The trade structure of the final products can be given by

$$S_t = \begin{bmatrix} s_t^{1,1} & \cdots & s_t^{1,p} & \cdots & s_t^{1,N} \\ \vdots & \ddots & \vdots & & \vdots \\ s_t^{r,1} & \cdots & s_t^{r,p} & \cdots & s_t^{s,N} \\ \vdots & & \vdots & \ddots & \vdots \\ s_t^{N,1} & \cdots & s_t^{N,p} & \cdots & s_t^{N,N} \end{bmatrix}, \text{ and } s_{j,t}^{pr} = f_{j,t}^{pr} \big/ q_{j,t}^{r} \text{ as } f_t^{pr} = s_t^{pr} \otimes q_t^r$$

(12)

According to the above definitions, a structure decomposition analysis disentangles the

changes in $EEPE_{r,t}$ and $EEPI_{r,t}$ over time t and quantifies how much a certain component has contributed. We distinguish the changes between at home (region r) and at abroad (here we mean the other regions in China without region r). The elements are emission coefficients, the trade structure of intermediate products, production technology, the trade structure of final products and the total final demands. For region r ,

$$w_t = w_t^{(r)} + w_t^{(-r)} \quad (13)$$

$$L_t = L_t^{(r)} + L_t^{(-r)} \quad (14)$$

$$T_t = T_t^{(r)} + T_t^{(-r)} \quad (15)$$

$$S_t = S_t^{(r)} + S_t^{(-r)} \quad (16)$$

$$q_t = q_t^{(r)} + q_t^{(-r)} \quad (17)$$

where $w_t^{(r)}$ only includes the elements of the emission coefficients at home and other elements are zeroes. Correspondingly, $w_t^{(-r)}$ represents the emission coefficients at abroad and other elements are zeroes. In the same way, we can split the matrix q and the matrix H . At last, we use this method to split the matrix T and matrix S ,

$$T_t^{(r)} = \begin{bmatrix} 0 & \cdots & T_t^{1,p} & \cdots & 0 \\ \vdots & \ddots & \vdots & & \vdots \\ T_t^{r,1} & \cdots & T_t^{r,p} & \cdots & T_t^{r,N} \\ \vdots & & \vdots & \ddots & \vdots \\ 0 & \cdots & T_t^{N,p} & \cdots & 0 \end{bmatrix}, \text{ and } T_t^{(-r)} = T_t - T_t^{(r)} \quad (18)$$

$$S_t^{(r)} = \begin{bmatrix} 0 & \cdots & S_t^{1,p} & \cdots & 0 \\ \vdots & \ddots & \vdots & & \vdots \\ S_t^{r,1} & \cdots & S_t^{r,p} & \cdots & S_t^{r,N} \\ \vdots & & \vdots & \ddots & \vdots \\ 0 & \cdots & S_t^{N,p} & \cdots & 0 \end{bmatrix}, \text{ and } S_t^{(-r)} = S_t - S_t^{(r)} \quad (19)$$

The relationships of EEPE and EEPI with these factors can be expressed as

$$EEPE^r = g^r(w^{(r)}, w^{(-r)}, L^{(r)}, L^{(-r)}, T^{(r)}, T^{(-r)}, S^{(r)}, S^{(-r)}, q^{(r)}, q^{(-r)})$$
$$EEPI^r = u^r(w^{(r)}, w^{(-r)}, L^{(r)}, L^{(-r)}, T^{(r)}, T^{(-r)}, S^{(r)}, S^{(-r)}, q^{(r)}, q^{(-r)}) \quad (20)$$

In the literature regarding SDA, it is important to note that, when there are many determination factors ($n \geq 5$), the number of all decomposition forms is n! and different procedures can lead to different results [1-4]. Since we have n=10, the number of alternatives will be 3,628,800. However, according to the research conducted by Dietzenbacher and Los (1998), the average of two polar decomposition forms can approximate the results of all possible decomposition forms. At the same time, several studies [3, 5, 6] have shown that method of two polar decompositions provides a good approximation of the overall results. Therefore, the two polar decomposition forms are also applied here, and they can be depicted briefly as follows. Decomposition is started by changing the first variable first, followed by changing the second and third variables, etc. to derive the first polar form. The second polar form can be derived in the opposite manner. In other words, decomposition starts by changing the last variable first, followed by changing the second-to-last variable and so forth. Based on the above decomposition method, after calculating the geometric average of g^r_{polar1} and g^r_{polar2}, the change in EEPE from *t* year to *t*-1 year can be obtained, and when the same procedure was performed on EEPI, the equations can be briefly expressed as:

$$\Delta EEPE^r_{t-1,t} = \frac{\Delta EEPE^r_t}{\Delta EEPE^r_{t-1}} = \sqrt{g^r_{polar1} \times g^r_{polar2}}$$
$$\Delta EEPI^r_{t-1,t} = \frac{\Delta EEPI^r_t}{\Delta EEPI^r_{t-1}} = \sqrt{u^r_{polar1} \times u^r_{polar2}} \quad (20)$$

References:

1. Ang B, Huang H, Mu A. Properties and linkages of some index decomposition analysis methods . Energy Policy. 2009, 37(11).4624-4632. doi:10.1016/j.enpol.2009.06.017
2. De Boer P. Additive structural decomposition analysis and index number theory: an empirical application of the montgomery decomposition. Economic Systems Research. 2008, 20(1).97-109. doi:10.1080/09535310801892066
3. De Haan M. A structural decomposition analysis of pollution in the Netherlands. Economic Systems Research. 2001, 13(2).181-196. doi:10.1080/09537320120052452
4. Dietzenbacher E, Los B. Structural decomposition techniques: sense and sensitivity. Economic Systems Research. 1998, 10(4).307-323. doi:10.1080/09535319800000023
5. Xia Y, Fan Y, Yang C. Assessing the impact of foreign content in China's exports on the carbon outsourcing hypothesis. Applied Energy. 2015, 150.296-307. doi:10.1016/j.apenergy.2015.04.028

6. Xu Y, Dietzenbacher E. A structural decomposition analysis of the emissions embodied in trade. Ecological Economics. 2014, 101(5).10-20. doi:10.1016/j.ecolecon.2014.02.015
